# Factors related to resilience for mid‐ to late‐career‐stage veterinarians: a qualitative study

**DOI:** 10.1111/avj.13453

**Published:** 2025-05-22

**Authors:** IF van Gelderen, SM Matthew, ML McArthur

**Affiliations:** ^1^ Sydney School of Veterinary Science The University of Sydney Camperdown New South Wales Australia; ^2^ College of Veterinary Medicine Washington State University Pullman Washington USA; ^3^ School of Animal and Veterinary Science The University of Adelaide Roseworthy South Australia Australia

**Keywords:** job demands, job resources, resilience, resilience strategies, veterinarians, well‐being

## Abstract

**Background:**

This study aimed to explore how resilience in mid‐ to late‐career stage veterinarians in Australia is shaped by interactions between job demands, personal and contextual resources, strategies and outcomes.

**Methods:**

Semistructured interviews with 17 mid‐ to late‐career stage veterinarians were conducted with questions modelled on prior research investigating veterinarians' experiences, teacher well‐being and resilience. The Job Demands‐Resources model guided thematic analysis.

**Results:**

Mid‐ to late‐career stage veterinarians described how resilience is shaped by interactions between (1) demands of the job, (2) resources available, (3) strategies used and (4) resilience outcomes. Job demands and job resources categories were sub‐divided into four and seven themes respectively according to whether characteristics were based on personal or contextual factors. Strategies were grouped into seven themes representing intentional ways that veterinarians made effective use of resources. Three themes in the Outcomes category represented positive outcomes that were enabled through dynamic interaction between demands, resources and strategies undertaken to realise outcomes.

**Limitations:**

This study did not explore relationships between perceptions of resilience and veterinary work type, practice location, age or gender. Interviews were conducted before the global COVID‐19 pandemic, and results do not reflect any associated psychological impacts from that event.

**Conclusion:**

Resilience in mid‐ to late‐career veterinarians is a dynamic process driven by the interplay of demands, resources, intentional actions and outcomes. Although personal resources can be developed over time, job demands are largely contextual. Effective management practices can mitigate challenges and foster resilience.

**Abbreviations**
Brief COPE: brief coping orientation to problems experienced
COREQ: consolidated criteria for reporting qualitative research
JD‐R: job demands‐resources
P: participant

Framed around the concept of resilience, current research acknowledges not only the difficulties that specifically relate to work as a veterinarian,^1^, ^2^, ^3^, ^4^, ^5^, ^6^, ^7^, ^8^, ^9^, ^10^, ^11^ but also the positive resources, contexts and adaptive responses that allow individuals and the profession to navigate these difficulties and continue to thrive.^12^, ^13^, ^14^, ^15^, ^16^, ^17^, ^18^, ^19^ Previous research indicates that issues affecting veterinarians are due to a variety of personal and work‐related factors, which in combination may contribute to a decision to leave the profession.^1^ The combined effects of workplace conditions along with personal stress are reported to contribute to high levels of moral distress and burnout in veterinarians.^10^, ^11^ These are issues that affect all veterinarians, including those who report being mentally healthy and have high levels of mental well‐being.^8^

A robust body of research investigating resilience exists for the teaching profession and underpins frameworks that have been used to describe resilience in veterinarians. The teaching profession is one with similar issues to those faced by veterinarians with respect to burnout, disengagement and attrition. Resilience is recognised as a way for teachers to be both effective and to positively respond to challenges.^20^ In research investigating the challenges faced by teachers,^21^ risk and protective factors were identified with the goal to provide a framework that promotes teacher retention as well as supports teachers to thrive in their chosen profession. The resources available to support teacher resilience are categorised as being personal or contextual, and strategies to achieve outcomes associated with resilience are described.^22^, ^23^ The capacity for resilience is recognised in this research to be a dynamic process that can vary based on personal, organisational and relational factors.^24^, ^25^

Resilience is a complex process that can vary over time and in different contexts. As such, perceptions of resilience will change based on the demands being faced and the resources available, and, as described in the teaching profession, these can vary based on the career stage.^19^ As such, capabilities required for success or satisfaction as a veterinarian may also have different emphases at different career stages. Previous research identified an emphasis on capabilities such as interpersonal skills and team fit as important for initial practice.^26^ These capabilities continue to be important for a sustained career, but an increased emphasis on other personal and contextual resources and strategies for resilience such as goal‐setting and continued learning has been identified for veterinarians in the later stages of their career.^26^ In a recent study comparing new graduate, mid‐ and late‐career stage veterinarians, when personal resources were taken into consideration, there were no differences in reported general resilience among the groups.^27^ However, there are no current studies that explore, in detail, the experiences of mid‐ to late‐career stage veterinarians that shape their capacity for resilience.

The Job Demands‐Resources (JD‐R) model is accepted as a valid framework for investigations conceptualising resilience and well‐being in different occupations.^28^, ^29^ Using this model, burnout and disengagement with work are described in terms of excess job demands and lack of job resources. The overarching goal is to identify predictors of factors that enhance well‐being and positive engagement with work. Widely used in investigations of well‐being in the teaching profession^30^, ^31^, ^32^ as well as in the veterinary profession,^1^, ^12^, ^33^ the JD‐R model is used to describe associations between job demands, job resources and outcomes. Descriptions of factors contributing to resilience across a range of contexts have been investigated in the veterinary profession using the JD‐R model^12^, ^13^, ^14^, ^34^, ^35^, ^36^ with several studies integrating personal resources into the JD‐R model used to conceptualise resilience. Building on existing research that has largely focused on studies of veterinary students and recent graduates, the JD‐R model is an appropriate framework for conceptualising resilience in mid‐ to late‐career stage veterinarians.

Given that resilience is a process that may vary over time and in different contexts, it is pertinent to understand how it is shaped not only for early career veterinarians, but also for those veterinarians in the mid‐to late stage of their career. The current study used qualitative research methods to gain an in‐depth understanding of factors related to resilience for mid‐ to late‐career stage veterinarians in Australia.^37^ The research question for this study was: How is resilience in mid‐ to late‐career stage veterinarians shaped by the dynamic interaction between job demands, personal and contextual resources, strategies and outcomes?

## Materials and methods

Semistructured interviews were conducted with mid‐ and late‐career veterinarians about factors related to resilience. Interview questions were modelled on previous research investigating veterinarians' experiences of professional practice,^38^ and teacher well‐being and resilience.^30^ Similar to other qualitative studies investigating well‐being in the veterinary profession,^1^, ^12^, ^33^ the JD‐R model was used as an overarching framework for the analysis.^28^ Reflexive thematic analysis^39^, ^40^ was used to organise and describe transcribed interview data, with coding for themes based on guidelines described by Saldana.^41^ That is, the researchers immersed themselves in the data by reading and rereading interview transcripts. Systematic open coding was performed to identify meaningful features in the data, and identification and organisation of themes in the data set was based on relevance as it related to the research question. Defining and naming of the themes within categories was informed by the theoretical principles of the JD‐R model.^28^ Reporting of this study was guided by the COREQ checklist for reporting qualitative studies.^42^ Approval to conduct this research was given by the University of Adelaide Human Research Ethics Committee, approval number H‐2017‐073.

### Participants

Mid‐career (6–15 years postgraduation) and late‐career (>15 years postgraduation) veterinarians in Australia completed a survey investigating self‐efficacy, coping strategies, general resilience and resilience in veterinary practice.^27^ The survey was administered for a larger study investigating the effect of demographic and psychological factors on resilience in Australian veterinarians. Included were survey questions related to four validated psychological measures, the Brief COPE, Brief Resilience Scale, General Self‐Efficacy scale and the Veterinary Resilience Scale–Personal Resources.^27^ At the end of the survey, respondents were invited to provide contact details if they wished to participate in an interview about factors related to resilience. Of the 800 usable responses, 710 identified as mid‐ to late‐career stage veterinarians.^27^ To gain breadth in understanding participants' experiences of resilience, purposive sampling based on survey outcome measures was used to select interviewees from amongst the respondents who expressed interest in being interviewed. A list of 47 participants flagged for follow‐up to participate in an interview were representative of those who scored highly for coping and resilience and those who scored low for coping and resilience based on the validated psychological measures used for the initial survey. Similar numbers of higher and lower scoring participants were invited to interview, and all veterinarians invited to interview accepted. Interviews continued until data saturation had been reached. Of the 17 participants interviewed, 7 were male and 10 were female. All participants were mid‐career or late‐career, and age at the time of the interview was between 28 and 70 years old (average age 44.5 years old). Interviews were conducted over a period of 5 months between November 2018 and April 2019.

### Interviews

Researchers 1 and 2 (IVG and SMM) interviewed participants by phone or videoconference. Participants were advised that the purpose of the interviews was to seek their own experiences and perceptions of resilience, and that there were no correct or incorrect answers. Questions explored what participants thought resilience was and how they approached maintaining it, personal and contextual factors contributing to resilience, and strategies used to adapt to changes in a professional context. Additional questions were asked about the importance respondents placed on resilience in veterinary practice and who they saw as responsible for helping them develop and maintain resilience. Interview duration was between 22 and 46 min, with audio recordings transcribed using an audio transcription service.

### Researchers

All researchers are academics in veterinary education and have experience with qualitative research methods. Researcher 1 (IVG) and Researcher 2 (SMM) are veterinarians with clinical practice experience. Researcher 3 (MLM) is a clinical psychologist.

### Analysis

For the first cycle of the coding process, all interview transcripts were read and then reread by Researcher 1 to identify and code words and short phrases that captured the meaning of the transcribed interview data.^41^ Codes were both descriptive and ‘in vivo’ codes taken directly from what a participant said in their interview. Researcher 1 then reviewed the initial set of codes to organise and group transcribed data that were similarly coded or shared common characteristics. Patterns were identified and codes were categorised into a preliminary structure that aligned to the Job Demands–Resources model. Themes and subthemes were identified within these categories.

Repeated cycles of coding were conducted to refine the coding structure. The provisional coding structure developed by Researcher 1 was reviewed and developed through an iterative process after discussions with Researcher 2 every 6–8 weeks over a 12‐month period. During these meetings, the coding frame was reviewed and interrogated by Researcher 2, and refinements were made to the coding structure to reflect the meaning of the data more accurately. These refinements involved minor rearrangements of codes and reclassification of data categories. Final revisions were made in response to comments from Researcher 3. Once themes were organised into a coherent pattern, Researcher 2 independently coded two transcripts to evaluate the applicability of the themes to the data set and to identify if additional codes were needed to fully represent the data. After refinements, a final codebook of themes and subthemes was created with definitions and illustrative quotes. In keeping with qualitative methods,^39^ the frequency of themes across the data set are not reported, as quantitative data of that nature does not indicate the relative importance of individual themes.

The final stage of analysis sought to determine levels of intercoder agreement, with Researchers 1 and 2 independently coding three (18%) interview transcripts. Transcripts for intercoder agreement were purposively selected due to the depth and breadth of participant responses and were different from the interviews previously coded by Researcher 2 when refining the coding frame. Before this stage, Researcher 2 marked the transcripts into meaningful segments or paragraphs of text so that both coders were coding the same units of text.^43^ Transcribed paragraphs were used as the unit of analysis, with consensual agreement analysed for paragraphs that were coded by both researchers.^1^, ^43^, ^44^ The average prediscussion agreement was 79% and postdiscussion agreement was 100%.

## Results

Veterinarians in mid‐ to late‐career stage described how their resilience in the profession was shaped by interactions between (1) demands of the job, (2) resources available, (3) strategies used and (4) resilience outcomes. Each of these four factors formed a category in the results. Subcategories of personal characteristics and contextual characteristics were identified for job demands and resources based on whether the characteristics within the category were based on personal or contextual features. Themes and subthemes were identified within each category or subcategory.

An overview of categories and themes is presented in Table 1. The final coding frame, including descriptions of categories, subcategories, themes, subthemes and sample statements, is presented in Table 2.

**Table 1:** The four categories and their themes for shaping resilience in mid‐ to late‐career veterinarians

Job demands	Job resources	Strategies	Outcomes
Personal Personal characteristics	Personal Personal attributes Self‐schema Prior experiences Support networks	Reflective practice Effective communication Help‐seeking behaviours Continuing professional development Setting boundaries Lifestyle behaviours Benevolent behaviours	Functioning effectively Job satisfaction Sustained engagement
Contextual Management Work‐life balance Work substance	Contextual Management Professional relationships Work substance		

**Table 2:** Final coding frame with sample statements for job demands, resources, strategies and outcomes shaping mid‐ to late‐career stage veterinarian resilience, including personal and contextual characteristics contributing to the dynamic interactions between these factors

Category	
Subcategory	
Theme	Description
Subtheme	Sample statements
Job demands	Job demands refer to job related risk factors, challenges, or potential threats. The demands include both contextual and personal characteristics that require effort for the individual and when excessive it can lead to exhaustion.^28^
Personal characteristics	Factors related to personal characteristics in an individual that may hinder resilience.
Personal characteristics	Personal characteristics that pose a challenge or risk in a veterinary workplace and may hinder resilience. P6: ‘…*if you're a personality that tends to be a bit of a negative kind of personality, you're always going to think about what you did wrong, rather than what you did right*.’ P9: ‘*I see some of my colleagues who haven't quite got over their perfectionist tendencies* [and] *struggle more with that*.’
Contextual characteristics	Factors related to the culture and conditions of the workplace that challenge workers and may hinder resilience.
Management	Management and organisational challenges that are linked to factors such as managing people, including clients and the structure and culture of the veterinary business.
Inflexible management	Rigid, uncompromising management practices that do not flexibly or quickly adapt and respond to change and individual variation. P5: ‘…*dealing with management and bosses often there's no outcome and that's very stressful …lack of consideration for personalities and circumstances*.’ P1: ‘*When I had that performance review, I essentially felt that I had to leave, immediately, even if I didn't have another job to go to, because they had given me a target that I couldn't feel I would be able to meet*.’
Poor organisational culture	Organisational factors that workers find challenging, which can include a lack of, or limited support from management and/or colleagues and teams. P5: ‘…*unless you're really supported by* [the] *clinic, I think you do just give up*.’ P14: ‘…*if I don't have support or backup, that's another huge aspect…lack of a supportive crew has certainly* [been] *the problem as far as things as that*.’
Difficult interactions	Perceived challenging interactions with relevant stakeholders, including issues such as stakeholders having unrealistic expectations, or being unreasonable or demanding, etc. Stakeholders include clients, other team members, practice owners, employees, etc. P8: ‘*Dealing with clients who are being difficult, for whatever reason. You know, if someone can't afford the treatment, that's fine, just tell me*.’ P16: ‘*…deal*[ing] *with resolving business stuff, business staff conflicts*.’
Transactional approach	These describe an approach where the primary goal of veterinary practice is seen as an exchange of service or product for money or meeting other targets relating to a business transaction. P5: ‘*I think it's focused on financials, and I guess also they're larger clinics and so they're less personal*.’ P1: *'I don't like feeling like a salesperson…pressure that there was with private practice to make money.'*
Inadequate recognition	These are challenges associated with a perception that work performed is not valued, including poor remuneration or lack of recognition from clients or the general public, etc. P6: ‘*…do*[ing] *after hours and things without getting paid*.’ P9: ‘*I just want to do what's right for the client and the family and when that's not appreciated, it's hard*.’
Work‐life balance	Challenges in achieving a balance between enjoyment at work with the ability to also enjoy aspects of life outside of work.
Discontinuity	These describe challenges associated with the loss of continuity and capacity to fully engage with work that arises from part‐time practice. P1: ‘*…that was stressful, too, because I was always in a rush and had to try to leave everything so that it could be handled the next day without me being there*.’ P5: ‘*When you go part‐time…you don't get interesting cases*.’
Extended work hours	This describes the challenges associated with extended work hours. This may be due to a requirement to do long workdays, after‐hours work, or a need to work more because of insufficient staff to meet demand. P15: ‘*I had a very old school boss who had very high expectations of long hours and hard work and no support. That's probably the biggest challenge, I think*.’ P12: ‘*After hours. It's the one thing that just still…It's like, I'm over that. But I don't have any resources to deal with it because the staff won't do after hours*.’
Work substance	This refers to the nature of work and level of expertise required of a veterinary professional.
Required skills and knowledge	This refers to the broad scope and superior level of required knowledge and skill needed for work as a veterinarian. P9: ‘*After I graduated from vet school…I found that challenging…as I just didn't have an adequate skill set to do justice to patient care*.’ P16: ‘*My mother is a GP, and she's just astounded about the workload. “What are you guys doing? You're doing orthopaedic surgery that normally a specialist would do in the human world*.” *We do every single medical discipline, we're doing it*.’
Uncertainty	This refers to the uncertain nature of veterinary work. It includes challenges associated with case complexity and negative outcomes, working with live animals and dynamic processes where information is often incomplete. P16: ‘*I ended up doing seven or eight extremely complex cases where I don't know what on earth is going on*.’ P9: ‘*We face challenges from a veterinary medical aspect, in terms of our patients* [they] *can't tell us what's wrong with them. Often what's wrong with them is not obvious based on history and physical exam and signalment and we then have to approach ‐ go down a diagnostic pathway, and sometimes we still end up without a diagnosis and a sick animal*.’
Demanding work environments	This describes the challenges associated with a demanding work environment. This may include, but is not limited to, intrawork issues such as rapid or pressured work pace and physically or emotionally challenging work environments. P15: ‘*I had 15‐minute appointments, but now where I'm working at the moment* [it] *really helps just to be able to take your time a little bit more…I'm a very slow‐worker; I'm not a fast‐worker like some vets*.’ P7: ‘*it does make it very emotional. I usually try to get through two to three euthanasias a day and usually by the third one I'm starting to wear down and become quite emotional afterwards*.’
Job resources	Job resources refer to health protecting aspects of the job. They include both personal and contextual characteristics and, when present, reduce the risk of burnout. When there is a lack of these resources it can lead to disengagement.^28^
Personal characteristics	Factors that are personal characteristics within an individual that promote resilience.
Personal attributes	Habits of personal behaviour that are personal strengths and are protective.
Positivity	Positive personal characteristics that are demonstrated in response to negative outcomes and dealing with adversity. It includes characteristics such as positive self‐talk, good humour and optimism. P17: ‘*a lot of it is to do with just positive self‐talk. You've got to talk yourself up, and you've got to make logic of it, and not get into negative sort of thought patterns, and let those progress*.’ P10: ‘*…having a sense of humour makes an enormous difference. If you can laugh at yourself, then you can laugh at anything*.’
Flexible	Personal characteristics that enable an individual to flexibly adapt, including when faced with uncertainty, change or as needed. P11: ‘*You've got to be a bit flexible. You've got to know when to let something go. If you're putting a whole lot of energy into it, and it's just not working out, you've got to reassess and say alright, maybe I should let this go.’* P5: ‘*If you can't change the situation, you just change your own situation*.’
Organised	The capacity to be organised and systematic. It includes the ability to manage time well. P8: ‘*But I think… you've just got to say, this is what I'm going to do, and organise yourself from there. A lot of it's about organisation*.’ P11: ‘*…timetabling things and… the support staff that you have, having them have clear roles so that they can be real good assistants to you*.’
Persistent	The capacity to be persistent, to continue engaging with work and life especially in response to challenges and adversity. P12: ‘*If you get knocked down, you get back up again. If you do that enough times, you can cope with just about anything*.’ P2: ‘*I think one of my most valuable assets is persistence*.’
Self‐schema	This is about ways that an individual perceives and feels about themselves. It is associated with aspects of emotional intelligence and are seen as protective when positive.
Self‐awareness	Being mindful or aware of own feelings, thoughts, actions and responsibilities. It includes having an understanding and acceptance of own feelings and capabilities and the impact that this has on others. P9: ‘*We need to be really honest with our clients about our own feelings and our own perceptions of what's going on*.’ P8: ‘*…recognise what triggers you towards anxiety or anger and work out what those triggers are and how you can avoid them or de‐personalise them is really important*.’
Self‐efficacy	An individual's belief in and awareness of their own capabilities and capacity with respect to their professional practice. P12: ‘*After enough years in practise and life even, if you're doing everything right then don't second‐guess yourself*.’ P9: ‘*I think that experience also helps…you get to that point where you know you've done everything possible and sometimes, you're not able to provide treatment that you know is ideal, or any treatment at all sometimes… I have enough experience as a clinician that I don't have any doubts in my clinical abilities, or ability to put the right team together, to provide veterinary care for an animal anymore*.’
Prior experiences	Personal experiences that presage an individual's experience as a veterinary professional. These prior experiences enhance an individual's ability to cope with stressors.
Foundational experiences	Prior experiences that were foundational or part of an individual's upbringing or childhood that occurred before becoming a veterinary student. They are seen to enhance or support an individual's understanding of, and ability to cope with, the stressors associated with professional practice. P14: ‘*If you have that good foundation, then you can only grow from there. Starting at primary school. Building resilience*.’ P12: ‘*My father was a vet (…) it was just a lifestyle which I was familiar with and that's why, even the after‐hours side of things (…) I just figure, oh well, I signed up for this, just keep toughing it out*.’
Education and training	Training or education gained while a veterinary student, whether at a university or elsewhere, that relates to supporting an individual's capacity to be resilient. P14: ‘*People need to, all the way through…to uni, having the skills or be shown the skills on how to do that* [resilience].’ P17: ‘*There's some fantastic, simple techniques out there, you know, how to manage other people and how to manage yourself. I think it should be part of the curriculum for…* [veterinary] *students*.’
Support networks	These are related to an individual's environment of support that is seen as protective and is linked to having the personal skill of help‐seeking behaviour.
Personal connections	This relates to relationships and interactions with personal networks that include family and friends. P17: ‘*It's really handy if you've got (…) other friends and family, whatever, that are supportive*.’ P3: ‘*I have vet friends, but also friends that aren't associated with vets from like a long time ago that are very constant*.’
Psychological support	Human, medical professional support networks such as psychologists, counsellors, psychiatrists and doctors, and the services they can provide. P6: ‘*I do have a psychiatrist who I see, and I am on medication*.’ P1: ‘*It's useful if the AVA have employee access programs, like you can access counselling sessions and stuff… not everyone's a member of the AVA* [so] *GPs and other ‐ beyondblue and other community support that's available to everybody*.’
Contextual characteristics	These are factors related to the culture and conditions of the workplace, professional relationships and the substance of work performed by a veterinarian that support workers and enhance resilience.
Management	These are management and organisational characteristics that support employee engagement.
Positive organisational culture	Organisational factors that enhance workplace culture and prosocial behaviours in the workplace. It includes fostering a sense of community and a supportive team environment. P9: ‘*…having a supportive workplace is important, and…I work very actively for my team to maintain that supportive workplace*.’ P4: ‘*I'm friends with everybody that's ever worked for me. I've still got nurses from 40 years ago who communicate with me. I get invited to all their weddings and I find that good. I've always tried to support my nurses and clients if they want to*.’
Size	The size of the veterinary practice team, the number of staff more generally and the number of other veterinary colleagues in the team. P3: ‘*I'm lucky I'm in a practice where we've got quite a few vets*.’ P9: ‘*The reason…that I work in a well‐resourced hospital with lots of amazing colleagues, is so that those challenges are minimised…I work with great colleagues that complement my skill set and so I know that I can provide the highest level of veterinary care*.’
Recognition	This relates to recognition given for work performed. It includes recognition in terms of remuneration, and/or acknowledgement of value to the organisation. P5: ‘*If you feel like you're valued, that actually goes a long way*.’ P7: ‘*If you're working long hours and you're being paid well, it's worth it and you feel like you're appreciated*.’
Professional relationships	These are industry specific aspects that support engagement within the profession.
Organised networks	This refers to formal relationships and organised networks that an individual has with the professional associations or networks. P11: ‘[The supports are] *professional associations like the Australian Veterinary Association, the professional associations like that have to move with the contemporary times. So, that has a role*.’ P13: ‘*Your messenger groups with people I went to university with. They can often give you a good perspective, because it's challenges they've come up against before. So that's really good*.’
Informal connections	This refers to informal relationships that an individual has with the peers, mentors or colleagues in the profession. P17: ‘*It's really handy if you've got supportive other people in the practice*.’ P2: ‘*I've got…a good circle of friends here, and colleagues here, at the place where I work. Those sorts of networks have been very valuable*.’
Work substance	Health protecting features that relate to the core work undertaken by a veterinary professional. It includes aspects related to the level of expertise and mastery developed by the time a veterinary professional is in mid‐ to late‐career stage, and to the pleasure derived from veterinary work.
Expertise	This relates to the individual's perceived levels of greater competence. It includes the level of expertise or mastery developed by the time the individual has reached the mid to late stage of their career. P13: ‘*I think experience helps*.’ P17: ‘*I was still learning ‐ you know, as you get more mature, you learn a lot more about managing yourself and managing other people*.’
Pleasure in work	This refers to work activities that veterinarians do that gives pleasure. It includes working with animals and being involved in interesting cases. P14: ‘*I did start another course that really allowed me to sort of reflect on what I enjoy in vet and in vet, I certainly enjoy the work*.’ P1: ‘*I feel like I'm able to focus on treating the patients which is what I wanted to do*.’
Strategies	Strategies are intentional, active and specific ways that make effective use of resources to navigate challenges that leads to resilience outcomes.
Reflective practice	This is a personal skill identified as protective. It involves critically reflecting on past actions and situations, navigating the emotions associated with these, and then adapting or responding to what was learned or discovered.^45^, ^46^ The capacity to engage in reflective practice indicates emotional intelligence. P8: ‘*Talk* [issues that hinder resilience] *through. Work through, again, is this personal, is it the situation that's the problem here*.’ P14: ‘*Talking about certain cases and communicating with my teammates or my group of colleagues and sort of saying this happened, what do you reckon? And then getting feedback… and then working on it that through that*.’
Setting boundaries	This is a personal skill identified as protective. It can include strategies such as setting limits and establishing separations between work life and personal life. P2: ‘*…just trying not to take work home with me, and I've always been reasonably successful with that. I'm the sort of person who can just shut work out and walk away from it*.’ P11: ‘*I realised I've got to balance things out a little bit…a balance between your work and how much effort was put into the work*, [and] *personal life*.’
Help‐seeking behaviour	This is a process where help is actively sought from others such as workplace mentors, peers, psychologists, family and friends. P6: ‘*I feel like… the person, as well needs to be able to sort of realise that they're struggling, and reach out for help, whether it's going to the doctor, or a psychologist, or a counsellor, or something*.’ P2: ‘*The ability to be able to talk to either experienced people in the profession, or mental health professionals, if you can see things are affecting your personal mental health, getting on top of them before they actually get worse*.’
Continuing professional development	This refers to activities intentionally undertaken to develop professional skills and knowledge after graduation, including clinical skills that are specific to performing work as a veterinarian and nontechnical skills such as communication, teamwork, leadership and decision‐making skills, etc. P7: ‘*I find that if I don't do a continuing education course, I become dissatisfied with the fact that I'm not continuing to learn something*.’ P9: ‘*A huge part of me feeling content in my daily work, is that I know that I have done a lot of training and I continue to work on my own knowledge and experience*.’
Lifestyle behaviours	These are protective lifestyle behaviours and activities that rejuvenate and provide balance between work and life.
Physical health	These are behaviours that are associated with maintaining physical health and include physical activities that the individual engages in outside of their professional life, behaviours linked to dietary choices and sleep behaviours. P8: ‘*I have a sport, which is incredibly important for maintaining my mental health. It's something that is very important that I spend time doing that and spend time basically away from vet doing the emotional recharge*.’ P4: ‘*Basically, diet, I think is pretty important. I exercise and because I have a good diet and good exercise and I'm not stressed out, I can actually sleep well*.’
Extracurricular activity	This relates to activities undertaken outside of work and include hobbies, such as gardening and cooking, and mindfulness activities such as meditation. P17: ‘*I developed a lot of other interests, which I mainly did on weekends. I sort of enjoyed my gardening, and you know, I used to work in the local reserve*.’ P2: ‘*I also do things that I find interesting and creatively satisfying. I do a lot of cooking, and I play music with friends, as well*.’
Effective communication	These are active ways of communicating with others that are open, and authentic or honest, including communication strategies that seek to understand and accommodate the perspective of others, etc. P17: ‘*Active listening, and not being defensive, and all that sort of stuff, is really important*.’ P9: ‘*I provide open and honest feedback to my staff, and I expect them to be able to do the same to me, and I think they're pretty good at that because they realise that they're allowed to give me feedback, even if it's constructive criticism*.’
Benevolent behaviours	These are behaviours that are characterised by a desire to help or benefit others. It includes actions that benefit people connected to the profession or workplace, and also other people who may not be directly associated with the individual or their work. P17: ‘*Yeah, you've got to do a bit of feel‐good stuff – Support a few charities, support some good causes, do a bit of voluntary work, you know, help other people*.’ P2: ‘*I've been a mentor in that program since 2005, so having that connection with the younger members of the profession, I've found incredibly valuable*.’
Outcomes	These are the positive outcomes that are enabled through the dynamic interaction between job demands and personal and contextual resources and the strategies undertaken to realise these outcomes.^15^, ^23^ The outcomes feed back into the interaction taking place between the job demands and job resources and influence strategies used.
Functioning effectively	This relates to the ability to function effectively both in work and life. That is, it refers to coping or functioning in ways that enable the individual to deal with stresses and adversity experienced in the workplace. P1: ‘*…ability to cope with the stresses and challenges of working in veterinary practice without, I suppose, mental or emotional affects*.’ P12: ‘*…continue to function effectively, without detriment to either work or your health*.’
Job satisfaction	This is an emotional state of fulfilment, care or satisfaction that is related to the way an individual feels about the work performed. P6: ‘*…be able to do the job and feel job satisfaction*.’ P14: ‘*Should… be able to have satisfaction of the job and not let the issues overcome them*.’
Sustained engagement	This describes a motivational state and refers to the capacity to be committed to engage with the work. P13: ‘*…go back to work the next day after you've had a particularly tough day*.’ P11: ‘*Resilience is the capacity to sustain a high level or an efficient level and a sustainable level and an enjoyable level. It's important so you avoid fall out*.’

**Figure 1:**
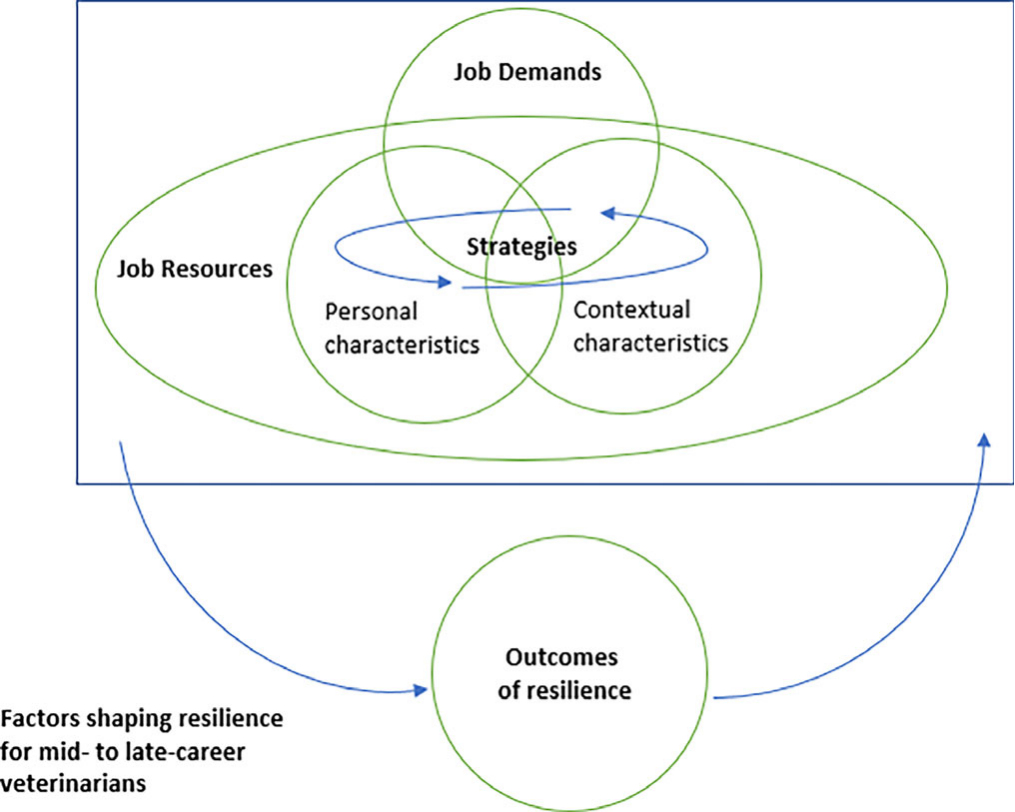
Job Demands‐Resources Model for conceptualising how resilience for mid‐ to late‐career stage veterinarians is shaped by the dynamic interplay between job demands, available resources for resilience, intentional strategies for resilience and the outcomes of these interactions, including personal characteristics that influence job demands and resources.

Dynamic interactions were described by participants between demands and resources and the ability, or not, to activate strategies so that resilience outcomes could be achieved. The interactions between personal and contextual job resources and demands, strategies and outcomes all work to shape resilience in mid‐ to late‐career veterinarians. This is represented in Figure 1 and examples are described in Table 3 with illustrative quotes. In the first example, access to the contextual job resource of informal connections (a subcategory within the category of professional relationships) was hindered by the contextual job demand of inflexible management. Strategies such as physical activity were described as helpful in supporting resilience in the second example but identified as only possible in the presence of contextual resources such as having more veterinarians due to the size of the clinic. Strategies such as extracurricular activity were described as more difficult to implement in the presence of contextual job demands such as extended work hours that impacted on work‐life balance (example 3). Further, in the fourth example the consequence of managing the job demand of extended work hours by moving to part‐time work led to the job demand of discontinuity in case management, removed a source of pleasure in work through doing surgery, limited enjoyment of the contextual resource of informal professional relationships within the workplace, and negatively impacted on the outcome of job satisfaction.

**Table 3:** Illustrative quotes demonstrating examples of dynamic interactions between factors contributing to resilience

Example	Illustrative quote
1	*I do like to debrief with other colleagues… maybe other veterinarians.…I find that quite helpful. In the past, I worked at a place where you sort of weren't allowed to say bad things about a client which was understandable…I think in that workplace they took it a bit too far. Because* [it] *sort of put that all, all the responsibility for making it a negative interaction back on me*. [P1]
2	*I've always been very active, but I did notice that when I first started and vet became all encompassing, that that was one of the first things to go and that I just didn't have time to exercise… I'm lucky I'm in a practice where we've got quite a few vets… I also negotiated with my new bosses… it just means I have more time to do that exercise as well*. [P3]
3	*Lots of hobbies, I guess. I want to always be learning and doing, just to keep busy…. When I was in mixed practice, it was definitely helping, but it was a lot harder to be able to juggle all those things and give each thing enough time. I'm finding it a lot easier now, now that, I guess, our work hours are more regular and yeah, more flexible*. [P13]
4	*I've really reduced my working hours considerably… I can choose the times that I work…. So trying to get that work life balance has then resulted in the challenges as far as job satisfaction. So now, I'm not getting case continuity or being able to … do enough of surgery, which I really enjoy, being able to work with that team environment on a regular basis, is probably the challenges I have as far as achieving my work life balance now*. [P14]

## Discussion

The findings of the current research illuminate how resilience for mid‐ to late‐career stage veterinarians is shaped by the dynamic interplay between job demands, available resources, intentional strategies and the outcomes of these interactions (Figure 1). This is consistent with the provisional definition of resilience in veterinary students, where resilience is conceptualised as an active, multifaceted process with outcomes that are adaptive and strengthened over time.^47^ The results build on the contextual focus of the JD‐R model by categorising key themes related to resilience in mid‐ to late‐career veterinarians as either personal or contextual for both job demands and job resources. Contextual demands or resources pertained to organisational and workplace specifics, whereas characteristics of individual veterinarians that may hinder or promote resilience were categorised as personal demands or resources. The results make a unique contribution in revealing how mid‐ to late‐career stage veterinarians enhance their well‐being and work engagement, framing resilience in terms of managing job demands, leveraging job resources and activating strategies to ensure that outcomes of these intentional actions can positively feed back into sustaining and enhancing resilience. For example, the outcome of achieving job satisfaction can positively feed back and enhance an individual's capacity to achieve pleasure in work, a contextual job resource described within the theme of work substance.

As shown in the results of the current research, balancing the demanding nature of veterinary work with other aspects of life is a challenge. This is evidenced by studies highlighting risk factors for mental health issues among veterinarians and students, such as lack of work‐life balance, heavy workloads and uncertainty.^15^, ^35^, ^48^ The substance of veterinary work itself, requiring high expertise and being emotionally and physically demanding, further compounds these challenges.^49^, ^50^, ^51^ Perfectionist traits, suggested to be common in veterinarians due to the profession's high entry standards,^52^ are associated with increased stress and moral distress which can lead to compassion fatigue.^53^, ^54^, ^55^, ^56^ However, these traits may not be as prevalent^57^ or maladaptive as once thought. Research investigating the effect of personality^57^, ^58^ suggests that traits such as neuroticism and depression are better predictors of occupational stress, and the risk of occupational distress diminishes over time.

Unhealthy coping mechanisms like depersonalisation may emerge as a way of coping with the challenges of veterinary practice.^58^ However, the current research demonstrates a robust set of positive strategies that can help to manage job demands effectively, leading to increased mastery. Similar trends are seen in teachers^59^ regarding teacher retention with reduced exhaustion over time.^13^ Aligned with the findings of research into teacher resilience,^23^ strategies identified in the current research include help‐seeking behaviour, setting boundaries to achieve work‐life balance, professional development activities, including those focussed on the development of reflection skills, and communicating effectively. As evidenced by our findings, however, tensions may exist between these strategies where, for example, setting boundaries was recognised as a useful strategy in achieving balance, but can also lead to a job demand with respect to discontinuity. Getting the balance right between these and other strategies is the challenge for individuals, employers and the profession more broadly.

Job resources, such as a positive work culture, professional relationships and the substance of veterinary work, play a crucial role in promoting engagement and preventing burnout among veterinarians. Described in veteran teachers^25^ and by participants in the current research, contextual resources tied to workplace conditions, including strong professional relationships and acknowledgment for work performed, support resilience. As the veterinary profession shifts towards a more corporatised environment, management must adapt to ensure these resources are maintained.^60^ The results of the current study demonstrate that mid‐ to late‐career veterinarians value networks and mentoring programmes (in the roles of mentee and mentor), which are essential for both early‐career transitions^16^, ^61^, ^62^ and sustained engagement. The inherent health‐protective effect from gaining pleasure in doing veterinary work,^12^, ^17^ combined with the mastery achieved in later stages of a career, contributes to job satisfaction and retention in a similar way to that seen in experienced teachers.^59^

The value placed on job resources by participants in the current study highlights the importance of understanding factors that encourage long‐term commitment to the profession and the intentional strategies undertaken to achieve this outcome. Employers, industry and representative professional organisations have a role to play in supporting engagement with personal resources and strategies as well as supporting social prescribing to manage stress. This type of social prescribing is where health professionals refer patients to social supports that typically promote the uptake of new hobbies or indeed activities such as volunteering.^63^, ^64^ Benevolence, such as volunteering and mentoring, was described by participants in the current research as supporting mental health and resilience,^65^ and we posit that it could be advantageous to broadcast these benefits more prominently and promote engagement in activities such as volunteering and being a mentor.

The results of this research highlight personal characteristics that contribute to job resources and promote resilience. These include positivity, flexibility, organisation and persistence. A positive self‐schema, or how individuals perceive themselves, is also crucial for maintaining mental health and engagement in work. Consistent with studies on teacher resilience,^19^, ^22^, ^23^ personal traits like self‐efficacy and emotional awareness are valuable job resources that can develop over time through education and experience. However, transitioning into the workforce can challenge these traits,^19^ emphasising the need for continuous support from workplaces and professional organisations in providing education as well as environments that enable a veterinarian to secure resources.^66^ The focus of this ongoing education should include areas related to emotional awareness and optimistic thinking^25^ as well as effective communication, reflective practice and ways to manage self. Additionally, personal networks, including family, friends, and access to psychological support, play a critical role in sustaining resilience. However, access to these personal resources for supporting resilience may be unequal due to factors like geographic location or minority status, making it essential for professional organisations and healthcare systems to ensure that support is accessible to all veterinarians.

Participants' comments in the current study affirm that challenges in veterinary work are closely linked to organisational culture and the nature of the job. Hostile workplace cultures can lead to frustration and decreased motivation,^67^ whereas challenging interactions with clients and external stakeholders can further hinder veterinarians' resilience.^49^, ^68^ Inadequate recognition from employers and clients exacerbates these challenges. The shift from small, privately owned practices to larger, corporate veterinary organisations has altered the professional landscape. Ensuring veterinarians have the skills required to work within or compete with corporate, well‐organised business cultures is an important consideration in veterinary curricula and continuing education.^60^ Effective communication is a vital skill for veterinarians in navigating this shift, especially since communication methods continue to evolve rapidly. Although communication is emphasised in veterinary curricula, ongoing professional development is crucial to keep up with progressive changes and maintain resilience. Other professional development opportunities include tailored programmes which can support veterinarians at various career stages, including transitions back to work after a break or preparing for retirement.^18^ By fostering a positive organisational culture and providing targeted support through continuing professional development, the veterinary profession can better equip its members to navigate the challenges of the evolving professional landscape.

**Figure 2:**
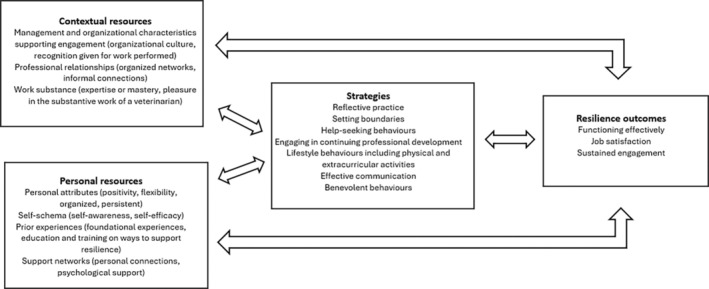
Positive contextual and personal resources for resilience, strategies for resilience and outcomes of resilience that interact to shape the process of resilience in mid‐ to late‐career veterinarians (based on Mansfield et al., 2016).

In summary, resilience in the veterinary profession is a dynamic process, shaped by the interaction between job demands, resources and intentional actions. The current research highlights the mutually reinforcing relationships between these factors, particularly in how mid‐ to late‐career veterinarians conceptualise resilience. Strategies to activate job resources, such as continuing professional development and effective communication, play a crucial role in this process. Job satisfaction, derived from the substance of veterinary work, such as caring for animals and engaging in interesting cases, enhances resilience by reinforcing motivation and expertise.^12^, ^17^ Sustained engagement, or the ability to continue working as a veterinarian, further strengthens the sense of mastery and expertise. Moreover, there is value in activating strategies, such as help‐seeking behaviour and extracurricular activities, to draw on both contextual and personal resources so that one can function effectively both in work and other aspects of life. The positive feedback loops identified in this research are ways in which positive action can be realised. Figure 2, modelled on diagrams of the resilience process in teachers^69^ and veterinary students^47^ signals the areas revealed in the current research that warrant focus and growth to realise positive change.

### Future research

The researchers acknowledge that there are current efforts to embrace suggestions aligned with those recommended here to support and maintain resilience in the veterinary profession. This is globally reflected in current veterinary school accreditation standards, but we suggest that future efforts and research interrogating these interventions are needed to investigate the effectiveness of what is being done to sustain this for veterinarians in the mid and late stages of their career. Future research investigating the impact of corporately owned veterinary practices on resilience in veterinarians is recommended, as this evolving operational framework may have benefits as well as hindrances with respect to resilience. Investigating resilience from a management perspective may give insight into potential benefits of resilience such as workplace efficiency and staff retention. Further, exploring possible differences in how resilience is conceptualised for owners and employed veterinarians, and between part‐time and full‐time veterinarians, is an area that warrants scrutiny and further investigation. Additionally, future quantitative research could explore causal relationships between the resilience factors described in this study.

### Limitations

Although participants were advised in a preamble script read before the interviews that the study was investigating their own experiences and that there were no right or wrong answers, it is possible that participants may have answered in ways that may be seen as socially desirable. Further, participants were selected based on whether they were interested in a follow‐up interview after completion of an online survey. Participants were not reimbursed for their time and their motivation to be involved in this study and willingness to give their time to participate may have created self‐selection bias. Further, interviews for this study were conducted before the COVID‐19 pandemic, which may have impacted factors associated with resilience in mid‐ to late‐career veterinarians in ways that are not reflected in the results of this study. This study did not seek to identify relationships between perceptions of resilience and other demographic data, such as type of veterinary work, location of practice, age or gender of participants. Although some of these factors were implied in participant responses, we recommend that future studies investigate differences based on demographics and across work contexts.

### Conclusion

In conceptualising resilience for veterinarians in the mid to late stage of their career, there is cause for hopeful optimism, provided we hear the message and act. Personal resources noted in the study are capacities that can be taught and developed over time. Moreover, job demands were largely contextual, and the framework presented here provides a roadmap for organising management practices in ways that overcome the inherent challenges associated with work as a veterinarian. As such, it behoves all leaders to assure that resilience factors are considered in management structures, professional development training and support. Further, training and education opportunities for veterinarians in practice should be targeted to strengthen job resources to effectively manage demands; that is focused on skills and knowledge for developing mastery and expertise and in nontechnical skills such as reflective practice and effective communication.

## Conflicts of interest and sources of funding

The authors declare no conflicts of interest or sources of funding for the work presented here.

## Data Availability

The data that support the findings of this study are available from the corresponding author upon reasonable request.
